# Selective Inhibition of Succinate Dehydrogenase in Reperfused Myocardium with Intracoronary Malonate Reduces Infarct Size

**DOI:** 10.1038/s41598-018-20866-4

**Published:** 2018-02-05

**Authors:** Laura Valls-Lacalle, Ignasi Barba, Elisabet Miró-Casas, Marisol Ruiz-Meana, Antonio Rodríguez-Sinovas, David García-Dorado

**Affiliations:** 1 Cardiovascular Diseases Research Group, Department of Cardiology, Vall d’Hebron University Hospital and Research Institute, Universitat Autònoma de Barcelona, Departament de Medicina, Barcelona, Spain; 2 Centro de Investigación Biomédica en Red Enfermedades Cardiovasculares (CIBERCV), Instituto de Salud Carlos III, Madrid, Spain

**Keywords:** Cardiovascular diseases, Acute coronary syndromes

## Abstract

Inhibition of succinate dehydrogenase (SDH) with malonate during reperfusion reduces infarct size in isolated mice hearts submitted to global ischemia. However, malonate has toxic effects that preclude its systemic administration in animals. Here we investigated the effect of intracoronary malonate on infarct size in pigs submitted to transient coronary occlusion. Under baseline conditions, 50 mmol/L of intracoronary disodium malonate, but not lower concentrations, transiently reduced systolic segment shortening in the region perfused by the left anterior descending coronary artery (LAD) in open-chest pigs. To assess the effects of SDH inhibition on reperfusion injury, saline or malonate 10 mmol/L were selectively infused into the area at risk in 38 animals submitted to ischemia-reperfusion. Malonate improved systolic shortening in the area at risk two hours after 15 min of ischemia (0.18 ± 0.07 vs 0.00 ± 0.01 a.u., p = 0.025, n = 3). In animals submitted to 40 min of ischemia, malonate reduced reactive oxygen species production (MitoSOX staining) during initial reperfusion and limited infarct size (36.46 ± 5.35 vs 59.62 ± 4.00%, p = 0.002, n = 11), without modifying reperfusion arrhythmias. In conclusion, inhibition of SDH with intracoronary malonate during early reperfusion limits reperfusion injury and infarct size in pigs submitted to transient coronary occlusion without modifying reperfusion arrhythmias or contractile function in distant myocardium.

## Introduction

The mechanisms of reperfusion injury are complex, but increased reactive oxygen species (ROS) production occurring short after flow restoration may play a key role^1^, modulating both cardiomyocyte hypercontracture and mitochondrial permeability transition pore (MPTP) opening^2,3^. Although low concentrations of both hydrogen peroxide and superoxide have been shown beneficial, mainly by activating prosurvival pathways, including those of preconditioning^4,5^, the massive ROS production occurring at the time of reperfusion is clearly detrimental. Indeed, ROS scavengers have been shown to be able to reduce myocardial infarct size in several animal models^6,7^. Furthermore, mutant mice overexpressing ROS scavenging enzymes, as superoxide dismutase, catalase or glutathione peroxidase, have reduced infarctions^8,9^, whereas those deficient in such enzymes are less resistant to reperfusion injury^10^. In addition, cardiac myocytes respond to H_2_O_2_ mimicking responses occurring after simulated ischemia-reperfusion, including a decrease in contraction amplitude and stimulation of the Na^+^-H^+^ exchanger^11^.

The potential sources of ROS in post-ischemic tissues include xanthine oxidase, NADPH oxidase, nitric oxide synthase, and especially the mitochondria^1^. Mitochondria might be, indeed, a major source of ROS generation during reperfusion in highly metabolically active tissues, like heart and brain. Electrons moving through the mitochondrial respiratory chain have the potential, during reperfusion, to leak and reduce O_2_ to superoxide (O_2_^−^), especially within mitochondrial complexes I and III^1,12^. Accordingly, complex I inhibition with amobarbital at the onset of reperfusion reduces infarct size in isolated rat or mice hearts submitted to ischemia-reperfusion in association with diminished ROS production^13,14^. In addition, different complex II or succinate dehydrogenase (SDH) inhibitors, including the reversible malonate, are able to modulate ROS production in isolated mitochondria in an ambivalent way: promoting superoxide generation at complex III under some conditions and attenuating ROS formation at complex I due to reverse electron transfer^15^. Diazoxide, a well known cardioprotective agent supposedly acting on mitochondrial ATP-dependent K^+^ channels (mitoK_ATP_), has been shown to inhibit SDH in isolated mice mitochondria^16,17^, which may contribute to its protective actions.

In this context, a previous study had shown that succinate, the natural substrate of SDH, accumulates in ischemic myocardium due to conversion from fumarate by reversal action of SDH^18^. In that work, authors suggested that upon reperfusion, resumption of forward SDH activity oxidizes accumulated succinate and induces a massive ROS production by reverse electron transfer from mitochondrial complex II to complex I^18^. Furthermore, we have recently demonstrated that reversible inhibition of SDH with malonate during reperfusion was also protective against infarction in isolated, Langendorff-perfused, mice hearts^19^.

However, malonate, as an inhibitor of mitochondrial respiration, may have toxic effects that would preclude its systemic administration in intact animals^20^. Thus, in this study we aimed to investigate the effect of selective intracoronary administration of malonate on infarct size in a clinically relevant pig model of transient coronary occlusion.

## Results

### Concentration-response curve to malonate under baseline conditions

**Figure 1:**
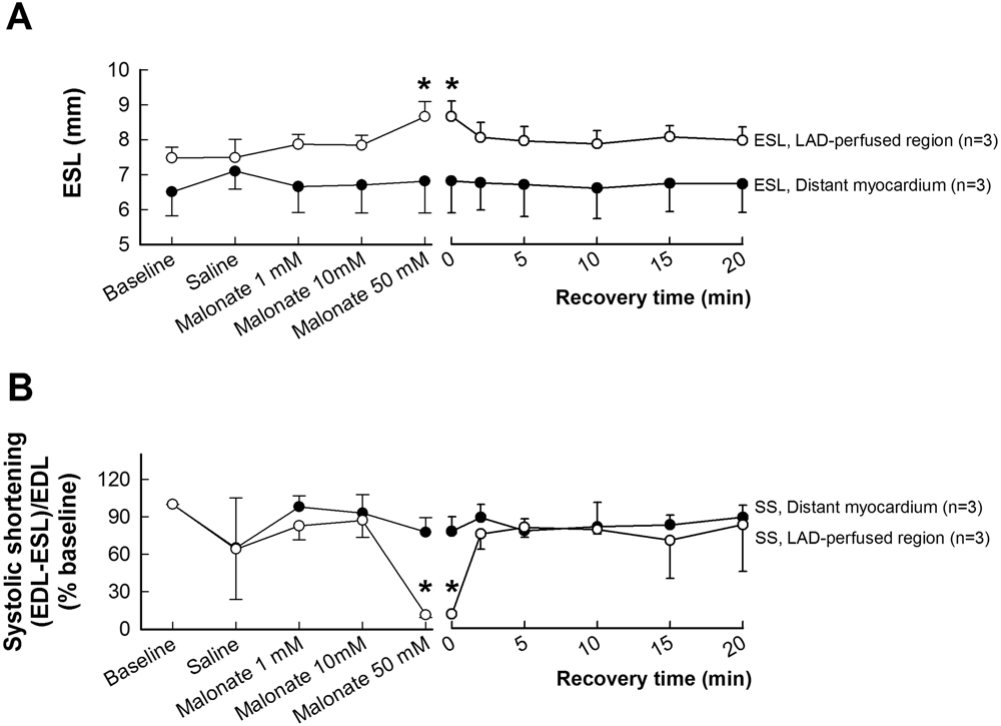
Effects of succinate dehydrogenase inhibition with malonate in myocardial function in the LAD-perfused region in pigs under baseline conditions. Changes in end-systolic length (ESL) (**A**) and systolic segment shortening (SS, expressed as percentage of baseline value) (**B**), measured by ultrasonic crystals placed in the distant myocardium and in the LAD-perfused area, during intracoronary infusion of saline or increasing concentrations of disodium malonate in pigs. Baseline SS values were 0.21 ± 0.04 in distant myocardium and 0.13 ± 0.03 in the LAD-perfused region. *(p < 0.05) indicates significant differences vs. the corresponding baseline value.

Neither saline nor malonate, at 1 or 10 mmol/L, induced significant changes in hemodynamics or regional myocardial contractility (Fig. 1). In contrast, infusion of 50 mmol/L of disodium malonate caused a significant increase in end-systolic length (ESL) at the end of infusion, in the apical area (Fig. 1A), receiving irrigation from the catheterized coronary artery, but not in the distant myocardium. Consequently, the increase in ESL induced a marked decrease in systolic segment shortening (SS) in the LAD-perfused region (Fig. 1B), indicative of reduced contractility. These changes in contractility recovered quickly and were not visible 2.5 min later (Fig. 1). Changes in other hemodynamic variables were negligible.

No ventricular fibrillation (VF) was observed at any malonate concentration tested. On the contrary, a single animal developed a VF during saline infusion.

### Malonate plasma concentrations by ^1^H-NMR (nuclear magentic resonance spectroscopy)

**Figure 2:**
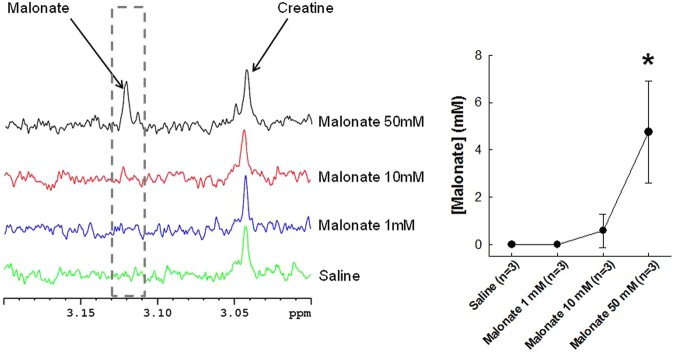
Nuclear magnetic resonance spectroscopy analysis of porcine plasma samples obtained from pigs treated with malonate under baseline conditions. (**A**) Representative ^1^H-NMR spectra from porcine plasma samples obtained after intracoronary saline infusion or after treatment with malonate 1, 10 or 50 mmol/L. (**B**) Plasma concentrations of malonate under each condition. *(p < 0.05) indicates significant differences vs. values obtained after saline infusion.

Intracoronary infusion of saline or disodium malonate at 1 or 10 mmol/L did not induce a discernible malonate peak on ^1^H-NMR spectra obtained from peripheral blood samples. In contrast, plasma samples obtained after treatment with 50 mmol/L of malonate showed an increased concentration of this metabolite, with a clear peak at about 3.12 ppm (Fig. 2).

### Ischemia-reperfusion injury

#### Hemodynamic variables, LAD coronary blood flow and regional myocardial function

**Figure 3:**
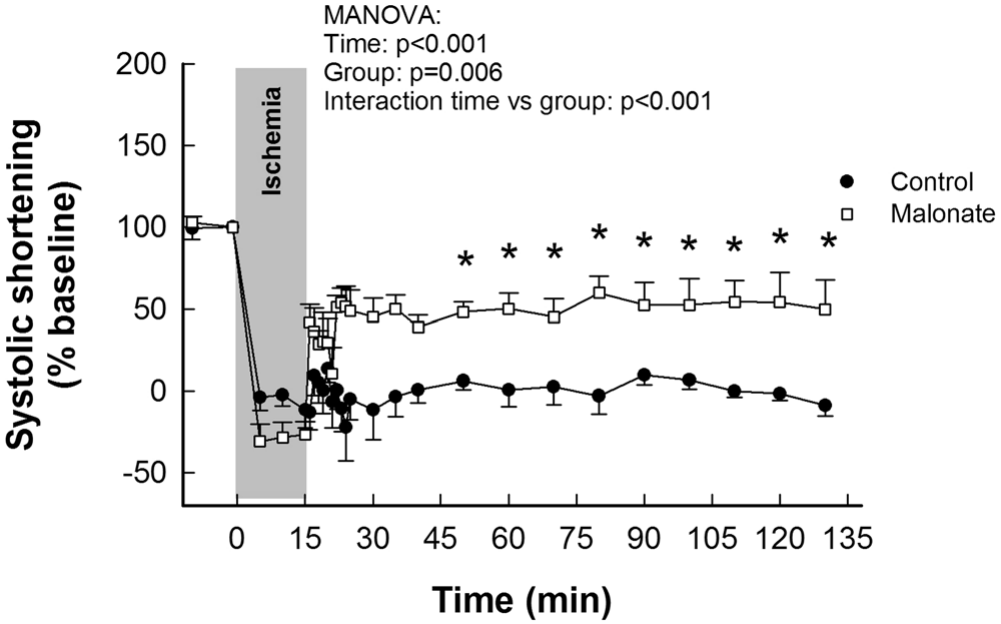
Myocardial stunning in the area at risk in pigs submitted to transient coronary occlusion and treated with intracoronary malonate during initial reperfusion. Changes in systolic segment shortening (SS, expressed as percentage of baseline value) in the area at risk in pigs submitted to 15 min LAD coronary artery occlusion followed by reperfusion, and treated with intracoronary saline or 10 mmol/L of disodium malonate. Baseline SS values were 0.29 ± 0.03 for control animals and 0.31 ± 0.06 for malonate-treated pigs. *(p < 0.05) indicates significant differences vs. the corresponding value in the control group.

Malonate significantly improved systolic segment shortening, during reperfusion, in the area at risk in animals submitted to 15 min of sub-lethal myocardial ischemia (Fig. 3), with no changes in hemodynamic variables. In contrast, in pigs submitted to 40 min of LAD coronary artery occlusion, no significant differences were observed between control and malonate-treated animals in the time course of any of these variables (Table 1, Supplementary Figure 1). Function of distant myocardium, as assessed by ultrasonic piezoelectric crystals was not affected by malonate treatment (Supplementary Figure 1).

**Table 1:** Values at baseline and at the end of ischemia and reperfusion for heart rate, aortic pressure (AP), left ventricular (LV) systolic and end-diastolic pressure (LVSP and LVEDP), LV (+) and (−) dP/dt, coronary blood flow at the LAD, and systolic shortening (SS) in the control and ischemic regions, in control pigs and in pigs treated with an intracoronary infusion of malonate 10 mmol/L.

	Control (n = 11)			Malonate 10 mM (n = 11)		
	Baseline	40′ ischemia	2 h reperf.	Baseline	40′ ischemia	2 h reperf.
Heart rate (beats/min)	80.56 ± 6.11	96.51 ± 7.99*	123.70 ± 8.28*	80.56 ± 6.12	90.45 ± 4.19*	98.32 ± 6.27*
AP (mm Hg)	126.46 ± 5.93	119.88 ± 7.59	125.09 ± 7.57	126.48 ± 5.93	116.30 ± 8.18	109.67 ± 8.69
LVSP (mm Hg)	129.03 ± 5.75	120.73 ± 8.98	129.43 ± 7.86	129.03 ± 5.75	117.18 ± 8.71	111.57 ± 8.95
LVEDP (mm Hg)	11.19 ± 1.43	12.67 ± 2.70	11.72 ± 2.51	11.19 ± 1.43	13.19 ± 3.61	11.73 ± 1.33
LV (+) dP/dt (mm Hg/s)	1607 ± 102	1651 ± 119	1723 ± 159	1607 ± 102	1762 ± 200	1400 ± 153
LV (−) dP/dt (mm Hg/s)	2553 ± 113	2348 ± 242	2578 ± 198	2553 ± 113	2031 ± 187	2056 ± 237
LAD flow (ml/min)	6.65 ± 1.65	0.00 ± 0.00*	11.24 ± 2.87*	6.65 ± 1.65	0.00 ± 0.00*	9.92 ± 1.36*
SS (control)	0.16 ± 0.03	0.15 ± 0.05	0.10 ± 0.03	0.21 ± 0.04	0.22 ± 0.04	0.16 ± 0.04
SS (ischemic)	0.18 ± 0.03	−0.05 ± 0.01*	−0.07 ± 0.02*	0.18 ± 0.03	−0.05 ± 0.01*	−0.06 ± 0.01*

*(p < 0.05) indicates significant differences vs. the corresponding baseline value.

#### Infarct size

**Figure 4:**
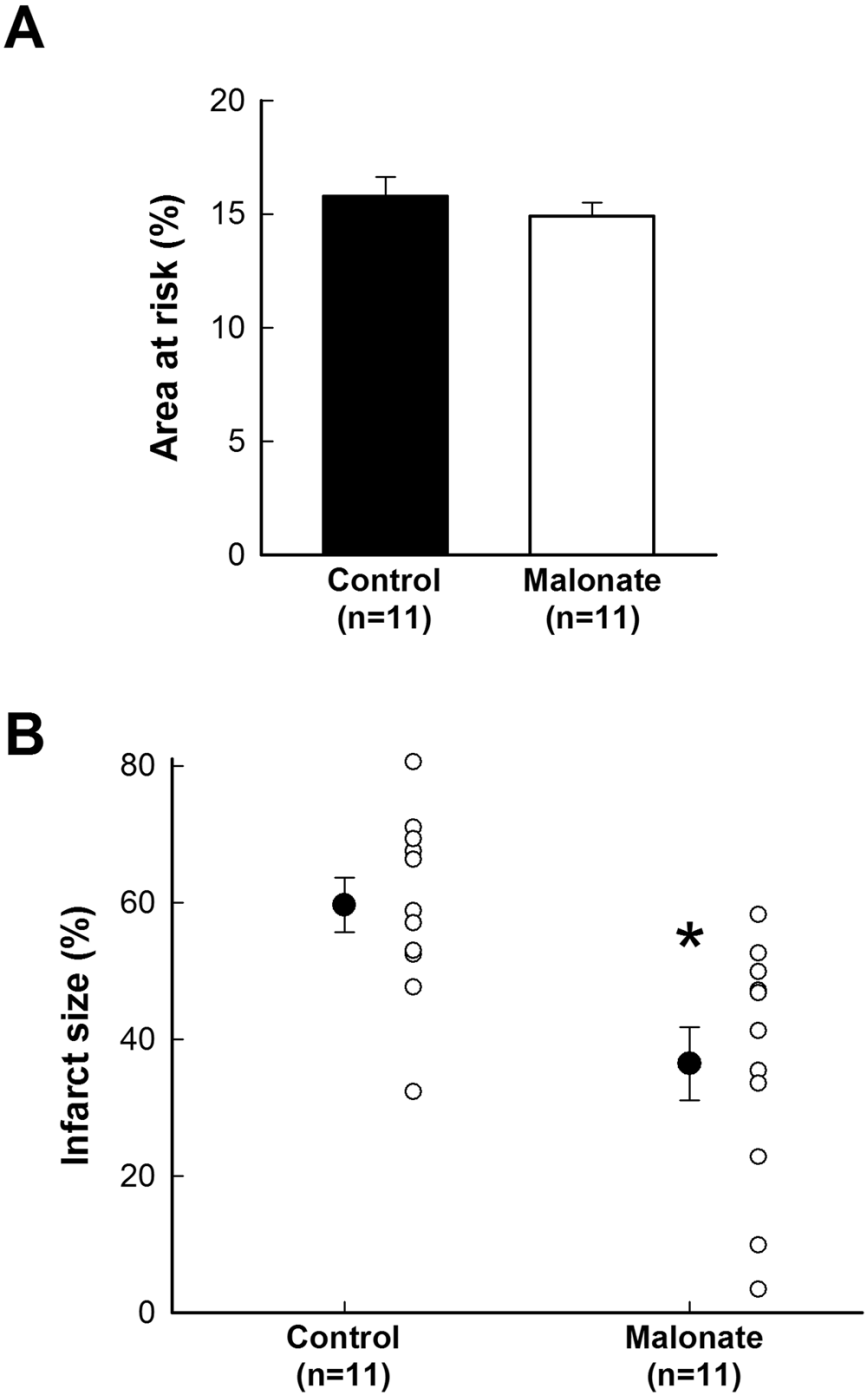
Effects of succinate dehydrogenase inhibition with intracoronary malonate during reperfusion on infarct size in pigs submitted to transient coronary occlusion. Size of the area at risk, expressed as percentage of the ventricular weight (**A**), in pigs submitted to 40 min of LAD coronary occlusion followed by 120 min of reperfusion, treated during initial reperfusion with an intracoronary infusion of saline (control) or disodium malonate 10 mmol/L. (**B**) Infarct size, expressed as percentage of the area at risk, in the same groups of animals. *(p < 0.05) indicates significant differences vs. control hearts (n = 11/group).

Infarct size in pigs submitted to 15 min LAD coronary artery occlusion was negligible in both control and malonate-treated animals. In contrast, in control pigs submitted to 40 min of LAD coronary artery occlusion, infarct size averaged 59.62 ± 4.00% of area at risk, and was significantly reduced by treatment during reperfusion with malonate 10 mmol/L (Fig. 4). No differences were observed between both groups in the size of the area at risk or body temperature (Fig. 4A).

#### Reperfusion arrhythmias

**Figure 5:**
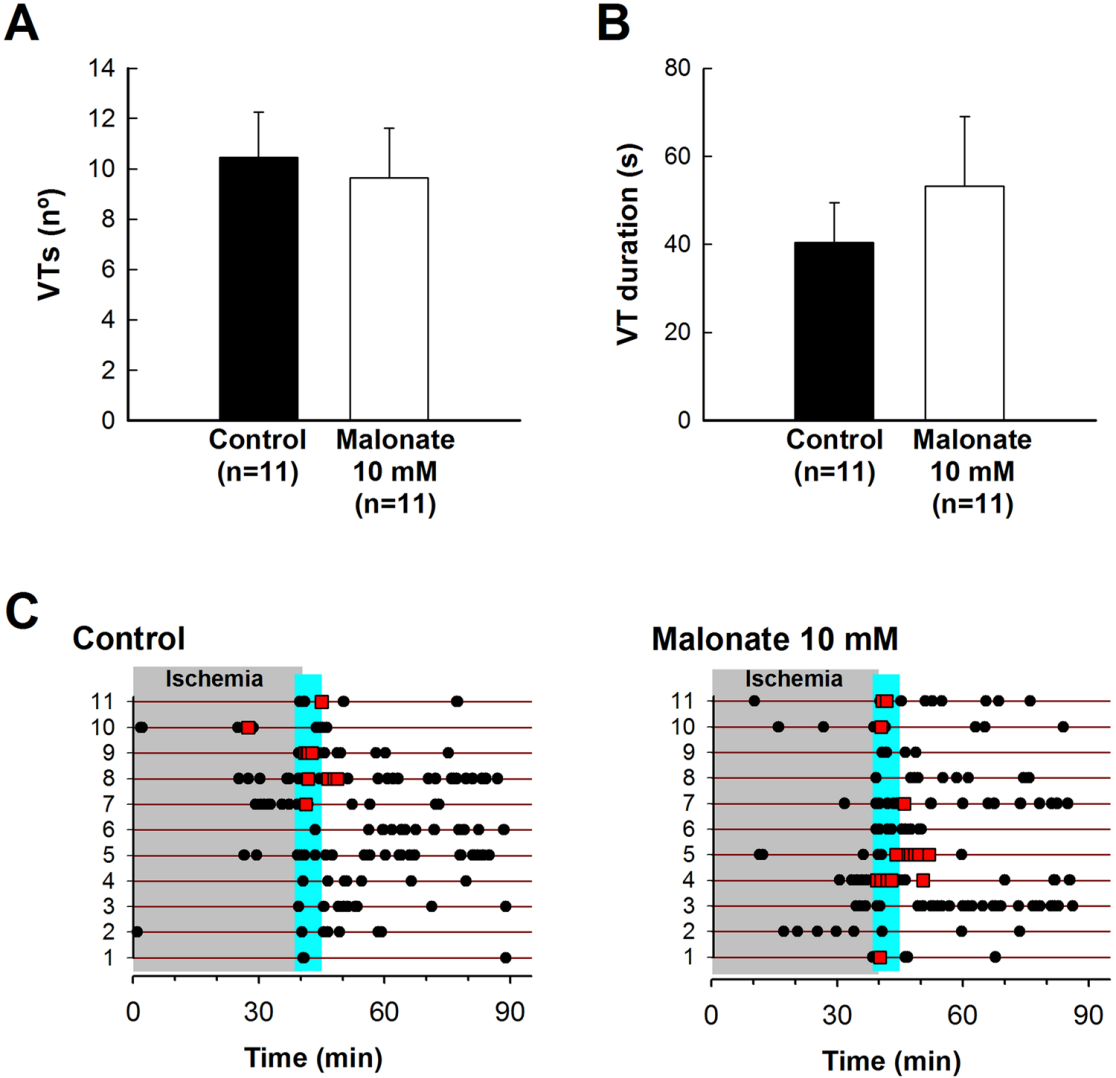
Incidence of reperfusion arrhythmias in pigs submitted to transient coronary occlusion and treated with intracoronary malonate during initial reperfusion. Total number of reperfusion ventricular tachycardias (VT) (**A**) and VT duration (**B**) in pigs submitted to 40 min of LAD coronary occlusion followed by 120 min of reperfusion, treated during initial reperfusion with an intracoronary infusion of saline (control) or disodium malonate 10 mmol/L. (**C**) Incidence of sustained ventricular tachycardia (VT, closed cercles) and ventricular fibrillation (VF, red squares) in the same group of animals. Blue square represents the duration of infusion.

No significant differences were observed in the total number of ventricular tachycardias (VT) or sustained VT, or in VT duration, during reperfusion after 40 min of myocardial ischemia (Fig. 5A–C). Similarly, 4 out of 11 control animals developed VF during reperfusion, an incidence that was not significantly different to that found in malonate-treated pigs (6 out of 11 animals) (Fig. 5C).

#### *Malonate and succinate myocardial concentrations after ischemia-reperfusion*

Myocardial metabolite concentrations were assessed in myocardial extracts obtained from additional experiments terminated after 5 min of reperfusion (n = 5/group). Two of the animals included in the group treated with malonate were excluded from the analysis due to poor reflow during the 5 min reperfusion period. In the remaining animals, we detected malonate only in tissue samples obtained from the area at risk from malonate-treated pigs (concentration of 7.77 ± 2.12 μmol/g wet tissue), with this metabolite being below detection limit in samples from control animals or in the distant myocardium of malonate-treated pigs. Succinate was increased in the area at risk of treated animals (Supplementary Figure 2).

#### ROS production in cardiac tissue during reperfusion

**Figure 6:**
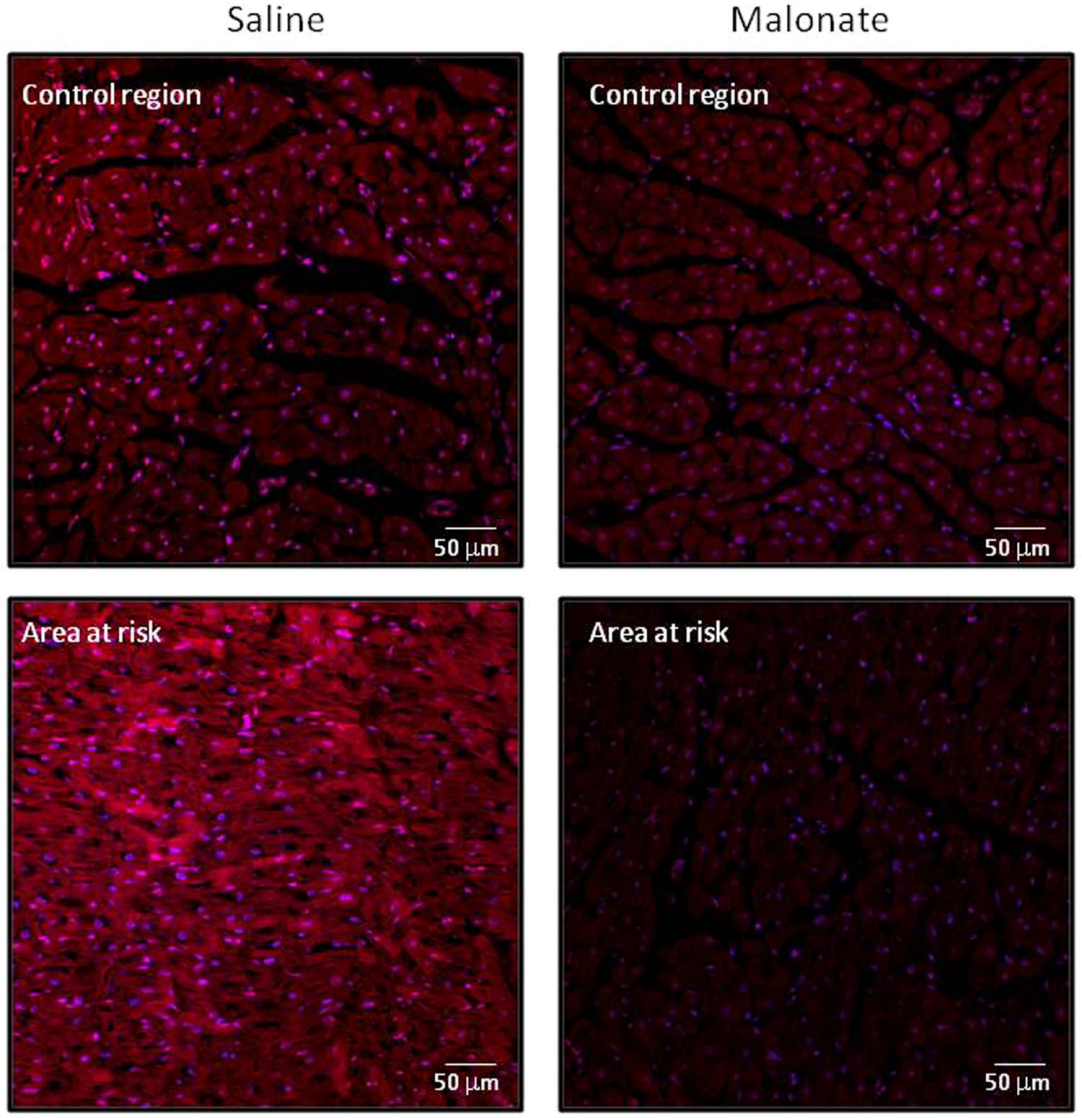
Malonate, given during reperfusion, reduces myocardial ROS production in porcine tissue samples. Figures show representative confocal images depicting MitoSOX staining (in red) (excitation wavelength 488 nm, emission wavelength 520 nm) in myocardial biopsies obtained 5 min after flow restoration, from both the area at risk and control region, in pigs treated with intracoronary infusion of saline (controls) or malonate 10 mmol/L. Nuclei are stained in blue.

Confocal fluorescence microscopy showed increased MitoSOX staining, 5 min after reperfusion, in the area at risk of saline-treated pigs compared with that observed in control myocardial regions. In contrast, malonate, given during initial reperfusion, attenuated this enhanced MitoSOX staining (Fig. 6).

#### Analysis of mitochondrial function

Oxygen consumption was assessed in the same animals used for the analysis of myocardial malonate and succinate concentrations, in which reperfusion was terminated after 5 minutes. As shown in Supplementary Figure 3, a trend towards a reduction in mitochondrial ADP-stimulated oxygen consumption was observed in the area at risk of both groups of animals.

## Discussion

This study shows that reversible inhibition of SDH with intracoronary malonate, given selectively in the area at risk, at the onset of reperfusion, is protective against reperfusion injury in a clinically relevant model of transient coronary occlusion. These effects were associated with reduced ROS production in the area at risk. The treatment did not modify the incidence of reperfusion arrhythmias or altered contractile function in distant myocardium. These results may be of interest for the development of treatments to prevent reperfusion injury in patients with ST-segment elevation myocadial infarction (STEMI).

Prevention of succinate accumulation or oxidation has been suggested as a new therapeutic target against myocardial reperfusion injury^21^. Succinate accumulates in ischemic tissues due to conversion of fumarate by reversal of SDH during ischemia^18,19^, and recovers its levels quickly during reperfusion. According to a recently proposed scheme, rapid succinate oxidation during reperfusion by forward SDH activity induces a massive ROS production by reverse electron transfer from mitochondrial complex II to complex I, and this oxidative stress leads to MPTP opening^18,19^. However, Andrienko and coworkers have recently suggested that conditions during early reperfusion are not favorable to reverse electron tranfer, and that the rapid decay in succinate occurring during reperfusion is due to efflux rather than oxidation^22^. According to this alternative hypothesis, MPTP opening would be triggered by factors other than ROS, including increased mitochondrial calcium concentrations, that in turn would cause a secondary increase in ROS, leading to more MPTP opening^22^. In this situation, the SDH inhibitor malonate would simply compromise the energy status of the cardiomyocyte, inducing protection^22^. However, our previous data indicate that malonate treatment only during reperfusion attenuates the rapid decay in succinate concentrations, suggesting that at least part of this recovery is due to oxidation^19^.

Chouchani and coworkers demonstrated that prevention of succinate accumulation during ischemia by pretreatment with malonate, a reversible inhibitor of SDH (or mitochondrial complex II), causes a reduction in infarct size in an *in situ*, open-chest, mice model of cardiac ischemia-reperfusion, effect associated with a reduction in ROS production^18^. Moreover, we have recently demonstrated that administration of the SDH inhibitor at the onset of reperfusion also reduces infarct size in isolated, Langendorff-perfused, mice hearts^19^. This effect was linked with a reduction in ROS production and with preserved mitochondrial function and increased calcein retention in isolated mitochondria, suggestive of reduced MPTP opening^19^. However, malonate might target mitochondrial respiration not only in the myocardium at risk, but also in other body cells. Indeed, malonate has been shown to exert deleterious effects, including cell death, in neuroblastoma-derived SH-SY5Y cells^20^. To avoid potential toxic effects, we selectively applied malonate into the area at risk.

Intracoronary drug administration selectively at the area at risk is clinically feasible and it has been previously used for the selective delivery of cardioprotective treatments, as adenosine, in patients with acute myocardial infarction^23,24^. In the present study we administered malonate selectively in the area at risk using a 2.8/2.5 F intracoronary infusion catheter, as previously described^25^. The concentration we chose to apply after ischemia, 10 mmol/L, was devoid of significant effects on systolic segment shortening under baseline conditions, and was not detectable in plasma from these animals. Selective infusion of 10 mmol/L malonate during initial reperfusion reduced ROS generation and infarct size in pigs submitted to 40 min LAD coronary artery occlusion and improved functional recovery in animals submitted to shorter periods of myocardial ischemia.

The beneficial effect of a cardioprotective treatment on infarct size does not warrant its clinical applicability to patients. Previous studies have demonstrated an increased incidence of VF in pigs treated with an intracoronary acid Krebs infusion (pH 6.4), despite it caused a reduction in infarct size^25^. Similarly, gap junction uncouplers, which reduce infarct size when given during initial reperfusion^26^, have been shown to have proarrhythmic properties^27^. However, this was not the case with the intracoronary administration of malonate at the onset of reperfusion in our pig model.

In addition, re-circulation of intracoronary administered malonate could have unwanted effects outside the area at risk. However, intracoronary infusion of malonate at the dose we found to be effective was associated to undetectable concentrations in distant myocardium and in plasma, and to no measurable effect on distant myocardium.

A wide number of therapeutic strategies have been suggested to limit infarct size in experimental models. However, none of them is so far part of standard clinical practice. This failure of translation can be due, at least in part, to the fact that most of these studies were conducted in young and healthy animals lacking comorbidities, comedications or risk factors, and to failures in the design of clinical trials^28,29^. In addition, considering that ischemia-reperfusion injury might be due to different mechanisms, targeting on individual objectives will unlikely result in reduced infarctions. In this context, a combination therapy may be an interesting approach to limit myocardial infarct size^30^, as has been described with a combination of remote ischemic conditioning and treatments modulating myocardial metabolism, including exenatide and glucose-insulin-potassium^31,32^. Malonate, if not individually, in combination with conditioning strategies^30,33^, may be a good candidate to reduce myocardial infarct size in patients with STEMI. Malonate has the advantage, over other tested compounds, that it is a natural metabolite that appears in animal tissues^34^, whose effects are reversible. Its main limitation, its possible toxic effects in other tissues, might be solved by an intracoronary route of administration.

In conclusion, our data demonstrate that selective, intracoronary, administration of malonate at the onset of reperfusion protects against myocardial infarction in *in situ* pig hearts, and that this effect is not associated with undesirable effects in distant myocardium or with an increased incidence of reperfusion arrhythmias.

## Methods

The present study conforms to the guidelines from Directive 2010/63/EU of the European Parliament on the protection of animals used for scientific purposes and the NIH Guide for the Care and Use of Laboratory Animals (NIH publications N°. 85–23, revised 1996, updated in 2011). This study was approved by the Ethics Committee of our institution (Comitè Ètic d’Experimentació Animal, reference number: 13/15 CEEA).

### Animals and instrumentation

Forty-one hybrid farm pigs (25–30 Kg, 12 h fasting) were premedicated with tiletamine-zolazepam (4–6 mg/Kg, IM) and anaesthetised with sodium thiopental (25 mg/kg, IV, followed by continuous infusion at 6–14 mg/Kg/h) and fentanyl (5 μg/Kg, IV, followed by continuous infusion at 3–6 μg/Kg/h), before intubation and ventilation. The left femoral artery and vein were cannulated for aortic blood pressure and blood sample monitoring, respectively. A midsternotomy was performed, and the pericardium opened, suturing its free margins to the borders of the sternotomy to cradle the heart. The left anterior descending (LAD) coronary artery was dissected free at its midpoint, below the first diagonal branch, and surrounded by an elastic snare. This dissection site was used for coronary ligature, whereas a second dissection site, located one centimeter distally, was used for blood flow measurements. The right carotid artery was also dissected, and a Judkins 8 F guiding catheter was introduced through it to catheterize the left coronary ostium using a 2.8/2.5 F Transit infusion catheter (Cordis). Arterial blood gases were monitored during the experimental procedure, and maintained within normal limits. Lead II of electrocardiogram was continuously recorded in a computer to monitor heart rate and ventricular arrhythmias. Left ventricular (LV) pressure and LV dP/dt and coronary blood flow were measured using a Millar SPR-350 Mikro-Tip Transducer catheter (Millar Instruments Inc., Texas, USA) and a Transonic Flowprobe (Transonic System Inc, New York, USA), respectively, as previously described^35^. At the end of the experiments, animals were sacrificed by a sodium thiopental overdose (100 mg/kg, IV).

### Regional myocardial function

Regional myocardial function was determined in a control region near the cardiac base (distant myocardium supplied by the circumflex coronary artery) and in the apical area (i.e., the area at risk in those pigs submitted to ischemia, supplied by the LAD) using two pairs of hemispherical polystyrene crystals inserted perpendicular to the LAD into the LV myocardium as previously described^25^. End-diastolic length (EDL) and end-systolic length (ESL) were identified in the recordings, and systolic segment shortening ratio (SS) was calculated from the equation SS = (EDL - ESL)/EDL^25^. End-diastolic measurements were taken at the point at which positive dP/dt begins to rise, and end-systolic dimensions were taken 20 ms before the nadir of the negative dP/dt^25^.

### Study protocols

All animals received a bolus of sodium heparin through the femoral vein (100 UI/kg), and a 2.5 F intracoronary infusion catheter (TRANSIT, Cordis Neurovascular Inc., Florida, USA) was advanced into the LAD, distally to the first dissection site, through the carotid artery guiding catheter.

#### Concentration-response curve to malonate under baseline conditions

To assess the effects of inhibition of succinate dehydrogenase on hemodynamics and myocardial function, saline or increasing concentrations of disodium malonate (1, 10 and 50 mmol/L, dissolved in saline) were administered to 3 pigs, through the intracoronary catheter, at a flow rate of 15 ml/min (37 °C), for 5 min. A rest period of 25 min was allowed between each infusion. Blood samples were obtained from a femoral vein at the end of each infusion, and plasma was separated by centrifugation at 1800 g, for 15 minutes, at low temperature (4 °C).

#### Ischemia-reperfusion and intracoronary malonate infusion

In order to evaluate functional recovery and myocardial stunning after malonate administration, six animals (n = 3/group) were submitted, after heparin administration and intracoronary catheter advancement, to 15 min of sub-lethal myocardial ischemia followed by two hours of reperfusion. Ischemia was performed by occluding the LAD coronary artery around the infusion catheter using the elastic snare. In these pigs, the two pairs of polystyrene crystals were placed in the area at risk. In addition, to investigate the effects of malonate on infarct size, 22 additional animals (n = 11/group) were submitted to a longer period of ischemia, of 40 min, that was followed by reperfusion as before. In all cases, animals were randomly divided in two groups. A control group received intracoronary saline for 6 minutes, beginning at 39 min of ischemia and lasting for the first 5 min of reperfusion, at a flow rate of 15 ml/min (37 °C), whereas malonate-treated animals received malonate at a concentration of 10 mmol/L.

Ten additional animals (n = 5/group), treated or not with malonate 10 mmol/L, were used to obtain tissue samples after 40 min of ischemia and 5 min of reperfusion, as described below.

### Nuclear magnetic resonance

Malonate plasma concentrations were analyzed by ^1^H-NMR in blood samples obtained from pigs treated under baseline conditions. Plasma metabolites were extracted using the methanol:chloroform method as described previously^36^. Plasma extracts were dissolved in 500 μl of deuterium oxide containing 1 mmol/L TSP (3-(Trimethylsilyl)propionic-2,2,3,3-d4 acid sodium salt), added as internal and chemical shift standard. ^1^H-NMR spectra were acquired on a vertical bore 9.4 T magnet interfaced to a Bruker Avance 400 spectrometer. Each spectrum consisted in the accumulation of 64 scans using a fully relaxed pulse-and-acquire pulse sequence with presaturation of the residual water signal.

Myocardial metabolite concentrations were also assessed in myocardial extracts obtained from additional experiments terminated after 5 min of reperfusion. Cardiac metabolites were analyzed by ^1^H-NMR spectroscopy in 10 additional pigs submitted to 40 min of LAD occlusion and 5 min of reperfusion, and treated or not with malonate 10 mmol/L during the last minute of ischemia and the 5 min of reperfusion (n = 5/group). Cardiac metabolites were extracted using the methanol:chloroform method as before.

### Reperfusion arrhythmias

Recordings were analysed for the incidence of ventricular tachycardia (VT) and ventricular fibrillation (VF) during ischemia and the first 50 min of reperfusion. VT was defined as four or more consecutive premature beats of ventricular origin. Sustained VT were those lasting more than 30 s.

### Area at risk and infarct size

At the end of the experiments, the LAD was reoccluded and the size of the area at risk and of infarction were determined by 10% fluorescein and 1% 2,3,5-triphenyltetrazolium chloride (TTC) staining, respectively, as previously described^37^. Area at risk was expressed as percentage of total ventricular weight, whereas infarct size was expressed as percentage of area at risk.

### Quantification of reactive oxygen species (ROS) production in cardiac tissue

ROS production was quantified in myocardial biopsies obtained 5 min after flow restoration, in 4 control animals submitted to 40 min of ischemia and in 4 pigs treated with malonate 10 mmol/L. Tissue specimens were obtained both from the area at risk and from a control, distant, myocardial region, and were immediately immersed in paraformaldehyde. Myocardial, paraformaldehyde-fixed, sections (5 μm) were incubated with MitoSOX Red (50 μM, Invitrogen) for 15 min (room temperature and darkness) to assess ROS production by confocal microscopy as previously described (excitation wavelength 488 nm, emission wavelength 520 nm)^19^. Nuclei were stained with Hoeschst 33342 (10 μg/mL).

### Analysis of mitochondrial function

Pig cardiac mitochondria were isolated from myocardial samples obtained at 5 min of reperfusion in the same 10 additional animals used for tissue metabolite quantification, as previously described^19^. Mitochondrial respiration was assessed with a Clark-type oxygen electrode (Hansatech, UK) at room temperature after addition of respiration substrates.

### Statistical analysis

Data are expressed as mean ± SD. Student’s t test or repeated measures ANOVA (MANOVA, mixed modeling with Greenhouse-Geisser correction) (for analysis of one intergroup and one intragroup variable), and Tukey post-hoc tests were used to assess differences between groups. Incidence of VF was analyzed by the Pearson Chi-square test. Non-parametric Mann-Whitney U test was used to assess differences in the number and durations of ventricular arrhythmias. Differences were considered significant when p < 0.05.

## Electronic supplementary material

Supplementary figures
