# Author Correction: Selective Inhibition of Succinate Dehydrogenase in Reperfused Myocardium with Intracoronary Malonate Reduces Infarct Size

**DOI:** 10.1038/s41598-019-42591-2

**Published:** 2019-04-17

**Authors:** Laura Valls-Lacalle, Ignasi Barba, Elisabet Miró-Casas, Marisol Ruiz-Meana, Antonio Rodríguez-Sinovas, David García-Dorado

**Affiliations:** 1 Cardiovascular Diseases Research Group, Department of Cardiology, Vall d’Hebron University Hospital and Research Institute, Universitat Autònoma de Barcelona, Departament de Medicina, Barcelona, Spain; 2 Centro de Investigación Biomédica en Red Enfermedades Cardiovasculares (CIBERCV), Instituto de Salud Carlos III, Madrid, Spain

Correction to: *Scientific Reports* 10.1038/s41598-018-20866-4, published online 05 February 2018

The original version of this Article contained an error in Affiliation 1, which was incorrectly given as ‘Cardiovascular Diseases Research Group, Department of Cardiology, Vall d’Hebron University Hospital and Research Institute, Universitat Autònoma de Barcelona, Barcelona, Spain’. The correct affiliation is listed below:

Cardiovascular Diseases Research Group, Department of Cardiology, Vall d’Hebron University Hospital and Research Institute, Universitat Autònoma de Barcelona, Departament de Medicina, Barcelona, Spain.

This error has now been corrected in the HTML and PDF versions of the Article and in the accompanying Supplementary Figures file.

