# Supplementary material for: Selective Inhibition of Succinate Dehydrogenase in Reperfused Myocardium with Intracoronary Malonate Reduces Infarct Size
Source: Sci Rep. 2018 Feb 5;8:2442. doi: 10.1038/s41598-018-20866-4 (PMC5799359; doi:10.1038/s41598-018-20866-4)
Supplement: Supplementary file 1 — Supplementary figures [file 41598_2018_20866_MOESM1_ESM.pdf]

SELECTIVE INHIBITION OF SUCCINATE DEHYDROGENASE IN REPERFUSED MYOCARDIUM WITH INTRACORONARY MALONATE REDUCES INFARCT SIZE.

Laura Valls-Lacalle [a,b], Ignasi Barba [a,b], Elisabet Miró-Casas [a,b], Marisol Ruiz-Meana [a,b], Antonio Rodríguez-Sinovas [a,b] *, David García-Dorado [a,b] *.

[a] Cardiovascular Diseases Research Group, Department of Cardiology, Vall d'Hebron University Hospital and Research Institute, Universitat Autònoma de Barcelona, Departament de Medicina, Barcelona, Spain.
[b] Centro de Investigación Biomédica en Red sobre Enfermedades Cardiovasculares (CIBERCV), Spain.

*** Corresponding authors:**

Dr. David Garcia-Dorado OR Dr. Antonio Rodríguez-Sinovas.
Grupo de Investigación en Enfermedades Cardiovasculares, Servicio de Cardiología, Vall d'Hebron University Hospital and Research Institute, Pg. Vall d'Hebron 119-129, Universitat Autònoma de Barcelona, 08035 Barcelona, Spain. Phone: +34 934894038, Fax: +34 934894032.

E-mails: dgdorado@vhebron.net
antonio.rodriguez.sinovas@vhir.org

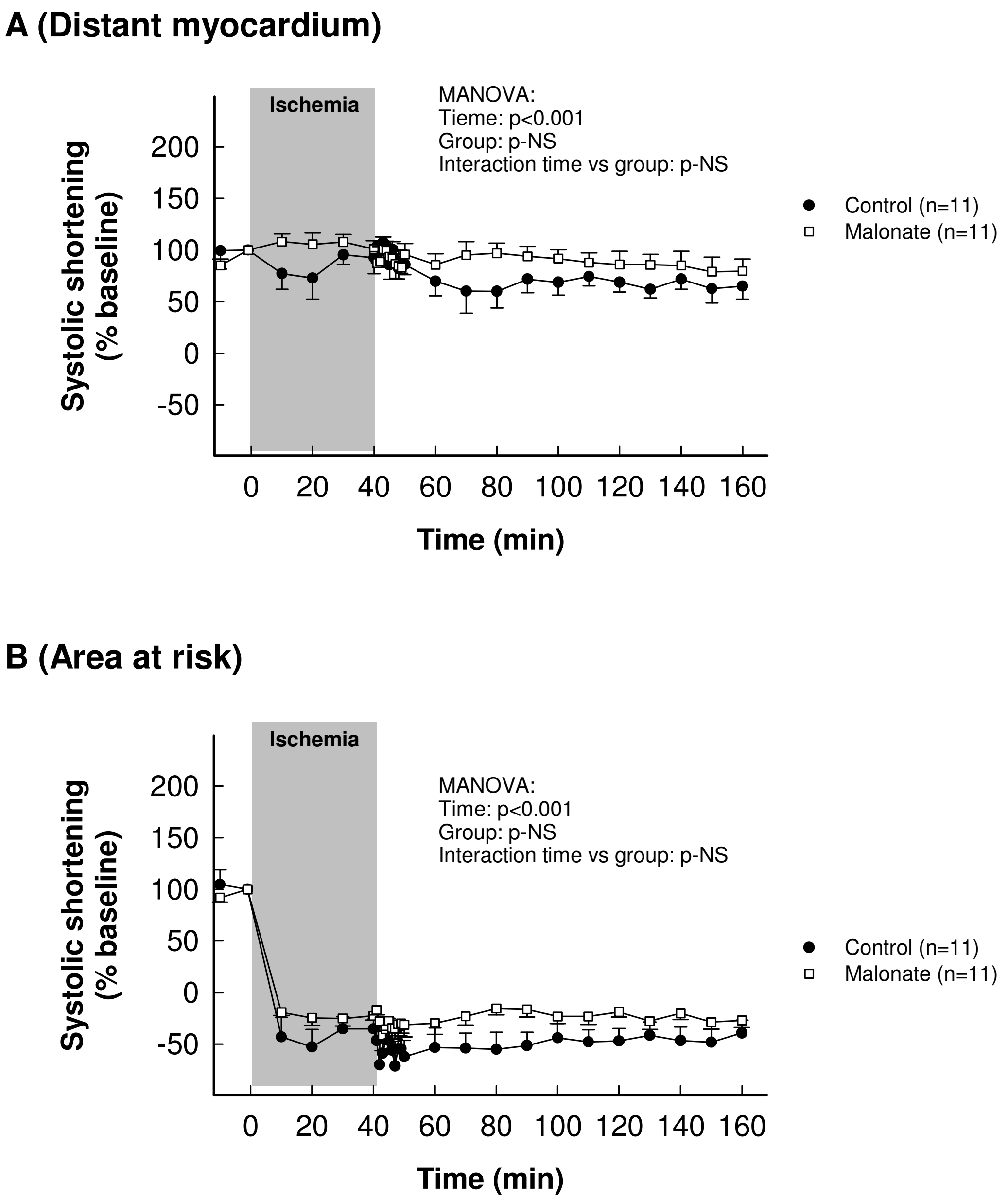

Supplementary Figure 1. Systolic segment shortening in the distant myocardium and in the area at risk in pigs submitted to 40 min LAD coronary artery occlusion followed by reperfusion, and treated with intracoronary saline or 10 mmol/L of disodium malonate. Malonate treatment did not modify systolic segment shortening in any myocardial region as compared with changes observed in control pigs.

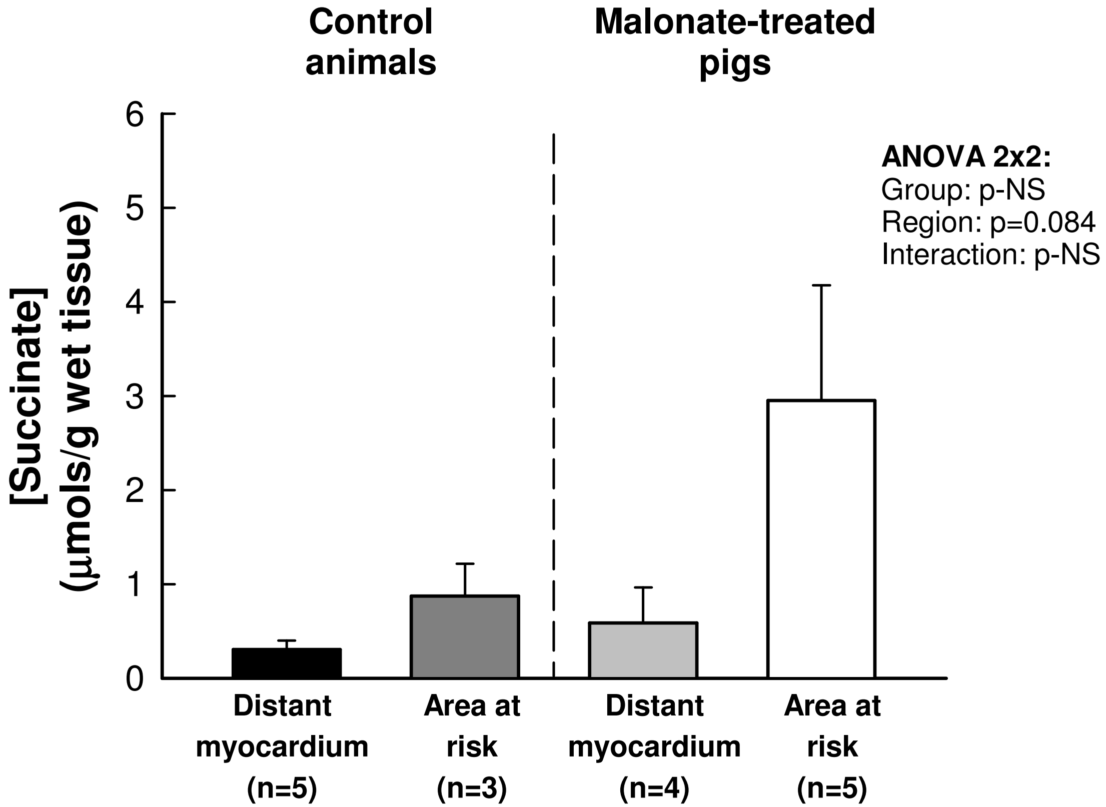

Supplementary Figure 2. Succinate concentrations in tissue extracts obtained from pigs submitted to 40 min LAD coronary artery occlusion followed by only 5 min of reperfusion, and treated with intracoronary saline or 10 mmol/L of disodium malonate. Succinate was significantly increased in the area at risk of treated animals vs. remaining groups pooled (2.95±1.22 vs. 0.54±0.16, Student's t test, $p=0.008$).

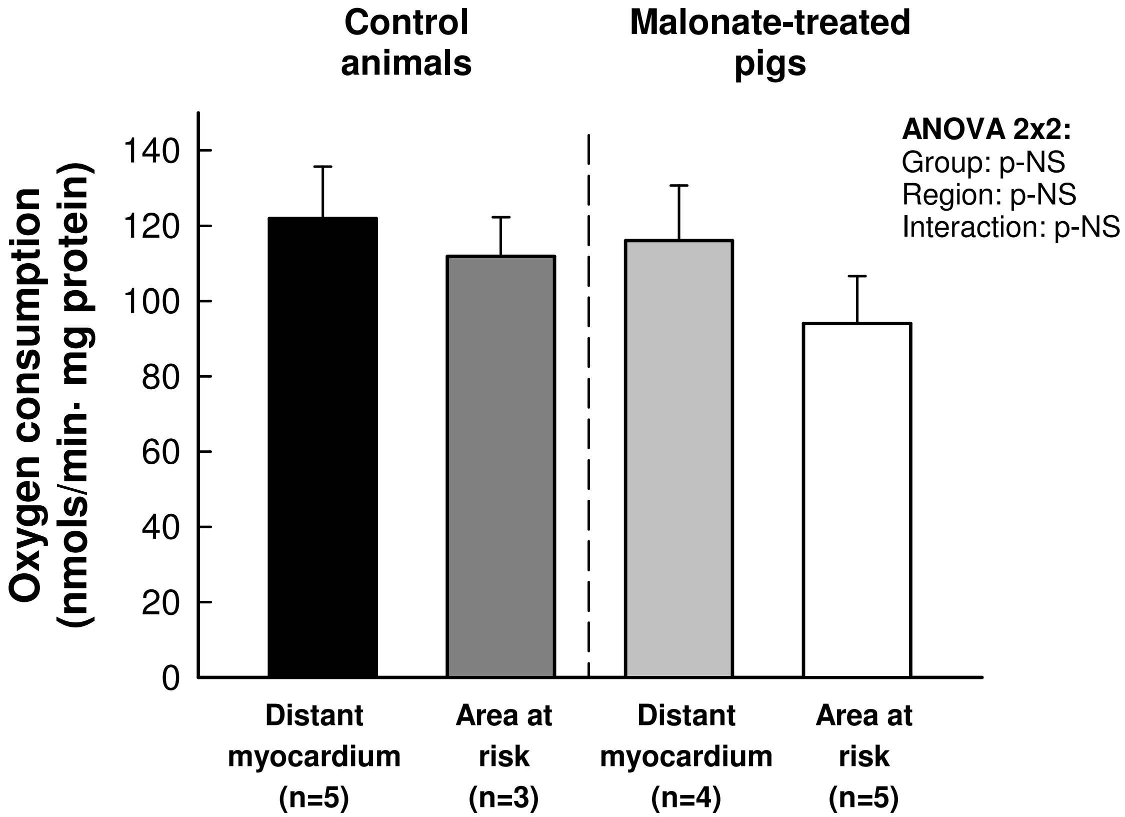

Supplementary Figure 3. ADP-stimulated oxygen consumption in mitochondria isolated from pigs submitted to 40 min LAD coronary artery occlusion followed by only 5 min of reperfusion, and treated with intracoronary saline or 10 mmol/L of disodium malonate. A non-significant trend towards a reduction in oxygen consumption was observed in the area at risk of both groups of animals, being slightly higher in malonate-treated animals.
